# Jasmonate Biosynthesis Across Bryophyte Lineages: Lessons from *Marchantia polymorpha* and Beyond

**DOI:** 10.3390/plants15132102

**Published:** 2026-07-07

**Authors:** Lucia Galassi, Francisco Medina-Paz, Guillermo H. Jimenez-Aleman

**Affiliations:** Department of Chemistry and Environmental Science, New Jersey Institute of Technology, Newark, NJ 07102, USA; lg422@njit.edu (L.G.); fm368@njit.edu (F.M.-P.)

**Keywords:** fatty acid, jasmonate, phytohormone, bryophyte, Marchantia, biosynthesis, plant defense, oxylipin

## Abstract

Jasmonates are lipid-derived phytohormones that regulate plant development and defense across the green lineage. Thus, understanding the intricacies of jasmonate biosynthesis and signaling is of paramount importance to improve crop yields and food safety. For the last 40 years, the canonical jasmonate biosynthetic pathway has been thoroughly dissected in angiosperms; however, only recent efforts have started to decode the alternative jasmonate biosynthetic networks that operate in bryophytes. In the nonvascular model *Marchantia polymorpha*, a *cis*-to-*iso* isomerization constitutes a key step in the formation of dn-*iso*-OPDA and Δ^4^-dn-*iso*-OPDA, the bioactive jasmonates recognized by a conserved COI1/JAZ co-receptor complex. Their biosynthesis depends on a uniquely expanded fatty acid repertoire that includes, alongside the canonical C_16_ and C_18_ omega-3 polyunsaturated fatty acids (PUFAs) found in angiosperms, substantial pools of eicosanoids such as arachidonic acid and eicosapentaenoic acid, essentially absent from flowering plants. Here we trace the jasmonate biosynthetic pathway in bryophytes step-by-step, from PUFA precursors production through lipoxygenase oxygenation and downstream reactions to the processing and catabolic modifications of bioactive compounds. By integrating current knowledge across bryophyte lineages, we identify mechanistic parallels and divergences relative to angiosperms, highlight key unresolved questions, and propose future directions for the field. Deciphering jasmonate biosynthesis in bryophytes is essential for reconstructing the evolutionary origins of jasmonate signaling and understanding how this pathway contributed to the successful colonization of land by plants.

## 1. Introduction

**Figure 1:**
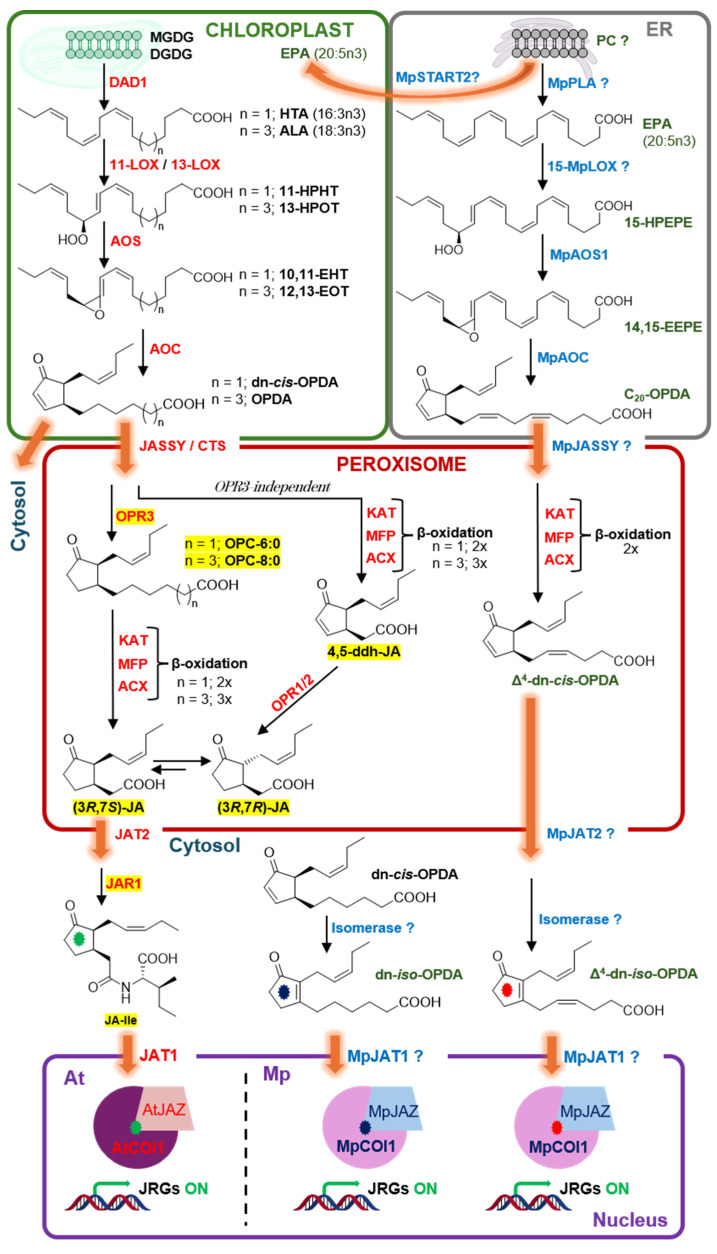
Comparative jasmonate biosynthetic pathways in *Arabidopsis thaliana* (At) and *Marchantia polymorpha* (Mp). Jasmonate PUFA precursors are synthesized in two distinct membrane compartments, the chloroplast pathway (top-left, green panel) and the ER (top-right, gray panel). ER-produced long-chain PUFAs (LCPUFAs) are proposed as *Marchantia*-specific jasmonate precursors. Subsequent processing happens in the peroxisome (center, red panel) and cytosol, before perception by a conserved co-receptor module occurs in the nucleus (bottom, purple panel). The orange curved arrow indicates a proposed translocation of EPA-containing lipids from the ER to the chloroplast mediated by MpSTART2 [25]. In Arabidopsis, OPDA and dn-*cis*-OPDA are reduced by OPR3 in the peroxisomes to produce OPC-8:0 and OPC-6:0, respectively. Rounds of β-oxidation yield (3*R*,7*S*)-JA, which can epimerize to the more thermodynamically stable (3*R*,7*R*)-JA. In *Marchantia*, the β-oxidation of jasmonates also occurs but appears to be context-dependent. *Marchantia* lacks JAR1 orthologs and ligand activation is achieved via a *cis*-to-*iso* isomerization by a putative isomerase. In the nucleus of Arabidopsis cells, JA-Ile bridges AtCOI1 and an AtJAZ to form a co-receptor complex that triggers JAZ degradation and de-repression of JRGs. This module is conserved in *Marchantia*, where MpCOI1 and the single MpJAZ are activated by dn-*iso*-OPDA or Δ^4^-dn-*iso*-OPDA as cognate ligands. Green, blue and red asterisks distinguish JA-Ile, dn-*iso*-OPDA and Δ^4^-dn-*iso*-OPDA ligands, respectively. ACX, Acyl-CoA Oxidase; ALA, α-linolenic acid; AOC, Allene Oxide Cyclase; AOS, Allene Oxide Synthase; COI1, Coronatine Insensitive 1; CTS, COMATOSE; ddh, didehydro; DAD1, Defective in Anther Dehiscence 1; DGDG, digalactosyldiacylglycerol; EPA, eicosapentaenoic acid; ER, endoplasmic reticulum; 10,11-EHT, 10,11-epoxy-hexadecatrienoic acid; 12,13-EOT, 12,13-epoxy-octadecatrienoic acid; 14,15-EEPE, 14,15-epoxy-eicosapentaenoic acid; 11-HPHT, 11-hydroperoxy-(*Z*,*E*,*Z*)-7,9,13-hexadecatrienoic acid; 15-HPEPE, 15-hydroperoxy-(*Z*,*Z*,*Z*,*E*,*Z*)-5,8,11,13,17-eicosapentaenoic acid. HTA, hexadecatrienoic acid; JA, jasmonic acid; JAR1, Jasmonate-Resistant 1; JASSY, chloroplast outer-membrane OPDA exporter; JAT1/2, Jasmonate Transporters 1 and 2; JAZ, Jasmonate ZIM-Domain; JA-Ile, jasmonoyl-L-isoleucine; JRGs, jasmonate-responsive genes; KAT, l-3-Ketoacyl-CoA Thiolase; LOX, Lipoxygenase; MGDG, monogalactosyldiacylglycerol; MFP, Multifunctional Protein; OPC, 3-oxo-2-(2′-pentenyl)-cyclopentane-acetic acid; OPDA, 12-oxo-phytodienoic acid; OPR, 12-Oxophytodienoate Reductase; PLA, Phospholipase; PC, phosphatidylcholine; START2, Steroidogenic Acute Regulatory Protein-related Lipid Transfer Domain Protein 2. ‘?’ denotes steps/enzymes proposed by sequence homology or biochemical inference but not yet experimentally confirmed. The orange straight arrows indicate translocation events.

Jasmonates are among the most evolutionarily ancient and functionally diverse signaling molecules in the plant kingdom, regulating processes ranging from pathogen and herbivore defenses to reproductive development and abiotic stress tolerance [1,2]. In angiosperms, the pathway is well understood and includes the release of omega-3 polyunsaturated fatty acids (n3-PUFAs) like α-linolenic acid (ALA, 18:3n3) from chloroplast membranes, sequential processing by Lipoxygenase (LOX), Allene Oxide Synthase (AOS), and Allene Oxide Cyclase (AOC) enzymes to yield *cis*-12-oxo-phytodienoic acid (OPDA), transport to the peroxisome, reduction by OPDA-Reductase 3 (OPR3), three rounds of β-oxidation to produce jasmonic acid (JA), and final conjugation by Jasmonate-Resistant 1 (JAR1) to isoleucine, generating the bioactive compound (3*R*,7*S*)-jasmonoyl-L-isoleucine (JA-Ile). The receptor complex comprising the F-box protein Coronatine Insensitive 1 (COI1) and a Jasmonate-ZIM-Domain (JAZ) transcriptional repressor perceives the ligand JA-Ile, leading to JAZ degradation and basic helix-loop-helix (bHLH) MYC2 transcription factor activation [3,4,5,6,7,8] (Figure 1).

Although core jasmonate signaling components are conserved in *Marchantia* (COI1/ligand/JAZ/MYC), several aspects are substantially different and their elucidation over the past few years has reshaped the understanding of jasmonate evolution. Seminal work has demonstrated that in *Marchantia polymorpha* (hereafter, *Marchantia*), dn-*cis*-OPDA and dn-*iso*-OPDA (positional isomers two carbons shorter than OPDA; throughout the text, we use ‘dn-OPDA’ generically when referring to both isomers and dn-*cis*-OPDA or dn-*iso*-OPDA is used when specifying the respective isomer) can bind the MpCOI1/MpJAZ co-receptor, not JA-Ile [9]. Upon ligand perception, proteasome-mediated MpJAZ degradation with the corresponding MpMYC-dependent transcriptional reprogramming proceeds analogously to angiosperms [9,10]. These discoveries were followed by the identification of an additional C_20_-PUFA-derived ligand, Δ^4^-dn-*iso*-OPDA [11,12], conserved Fatty Acid Desaturases (MpFADs) involved in the synthesis of dn-OPDAs [13,14], and the demonstration that dn-*iso*-OPDA amino acid conjugates, produced via the *Marchantia* Gretchen Hagen 3A (MpGH3A) enzyme, act as inactivating catabolites [15], in striking contrast to the activating role of JA-isoleucine conjugation in angiosperms. Broader phylogenetic and metabolic profiling subsequently showed that dn-*iso*-OPDA is the predominant stress-induced hormone across the bryophyte lineage and early-diverging lycophytes, while JA-Ile gained functional relevance within the lycophytes (with *Selaginella* representing a transitional state that accumulates both, dn-*iso*-OPDA and JA-Ile), in coordination with OPR3 and JAR1 enzyme evolution [9,16,17] (Figure 1).

The liverwort *M. polymorpha* has emerged as the leading model for studying these questions, owing to its basal phylogenetic position, haploid genome, and low gene redundancy. Its genome encodes single-copy orthologs of most jasmonate signaling components, and robust CRISPR/Cas9 tools have enabled unambiguous assignment of biosynthetic functions to individual enzymes [18,19,20]. While we draw on comparative data from mosses (particularly *Physcomitrium patens*) and, where available, hornworts to provide a broader bryophyte perspective, the depth of mechanistic and biochemical coverage throughout this review necessarily reflects *M. polymorpha* as the organism for which the most complete genetic, metabolic, and biochemical dissection of the jasmonate pathway has been achieved to date. This review is organized to follow the pathway sequentially. We first describe the PUFA precursor landscape (Section 2), then progress through the enzymatic steps LOXs (Section 3), AOSs (Section 4), and AOCs (Section 5) before covering the downstream *cis*-to-*iso* isomerization, amino acid conjugation, potential omega oxidation (hydroxylation), and β-oxidation reactions (Section 6); we conclude with an integrated assessment of key open questions (Section 7). Moreover, we identify analogies, parallelisms, and divergences with vascular plants, taking *Arabidopsis thaliana* (hereafter Arabidopsis) as the key model organism. Several comprehensive reviews have addressed jasmonate biosynthesis and signaling in angiosperms [7,8,17,21,22,23,24], and the present manuscript does not aim to duplicate that coverage. Instead, this review makes four specific contributions. First, we provide a step-by-step mechanistic account of the complete bryophyte jasmonate biosynthetic pathway, integrating the recently characterized C_20_-LCPUFA branch as an evolutionary novelty with no counterpart in angiosperms. Second, we discuss the *cis*-to-*iso* isomerization as a key ligand-activation step in *Marchantia*, recognizing the identification of the responsible enzyme as a critical unresolved gap in the pathway. Third, we highlight the deactivating role of MpGH3A-mediated amino acid conjugation in bryophytes—a functional inversion relative to the activating JA-Ile conjugation in angiosperms—with significant implications for understanding the evolutionary trajectory of jasmonate signaling. Fourth, we present an integrated comparative framework that traces mechanistic parallels and divergences between bryophytes and angiosperms at each biosynthetic step and systematically identifies the experimental priorities most likely to resolve outstanding questions. Collectively, this synthesis is intended to serve as a reference resource for researchers working on jasmonate evolution, oxylipin metabolism, and the biology of early diverging land plants.

## 2. Omega-3 PUFA Precursors: Fatty Acid Desaturation and the Unique Lipid Profile of Bryophytes

### 2.1. The Role of MpFAD5, and HTA as the Primary PUFA Precursor for dn-iso-OPDA Production

A specific and biochemically important Δ^7^-desaturase, MpFAD5, was identified as the key enzyme responsible for the biosynthesis of HTA in *Marchantia*. Stable isotope feeding experiments using deuterium-labeled HTA (d6-16:3n3) confirmed the rapid conversion of HTA-derived intermediates into dn-OPDA isomers without passing through OPDA [9]. Genetic knockout of Mp*FAD5* nearly abolishes dn-OPDA accumulation after mechanical wounding [13]. MpFAD5 is orthologous to AtFAD5, an Arabidopsis enzyme known to synthesize 16:1n9 (Δ^7^-palmitoleic acid) attached to the *sn*-2 position of monogalactosyldiacylglycerols (MGDG) in the plastids [26]. MpFAD5 functions as an n9-FAD acting on palmitic acid (16:0) to introduce the first unsaturation that ultimately leads to HTA via the evolutionarily conserved and concerted action of MpFAD5, MpFAD6 (a Δ^12^-desaturase; see Section 7 for its uncharacterized role), and FAD7 (*Marchantia* encodes a single n3-FAD ortholog in the chloroplast, in contrast to the two paralogs FAD7/8 of Arabidopsis) acting on MGDG lipids [13,26]. Genetic studies employing Mp*fad5* mutant plants confirmed that HTA, not OPDA β-oxidation, is the dominant source (>90%) of dn-OPDAs in *Marchantia* [13]. Importantly, while Mp*fad5* mutants showed only minor defects in jasmonate-regulated transcription and anti-herbivore defense compared with wild type (WT), the identification of MpDES6 operating in parallel to produce LCPUFA-derived jasmonates (C_20_-OPDA and Δ^4^-dn-*iso*-OPDA) explained this surprising observation [11]. This redundancy between the HTA-derived and EPA-derived jasmonate pools is a fundamental feature of bryophyte jasmonate biology with no counterpart in angiosperms [11].

### 2.2. The Canonical C_18_ and C_16_ Fatty Acid Pool

**Figure 2:**
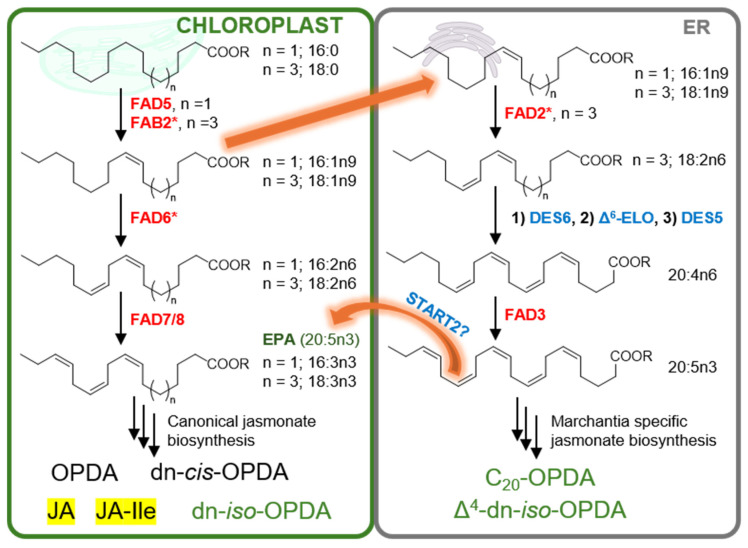
Biosynthesis of the jasmonate PUFA precursors in *Marchantia* and Arabidopsis. The two pathways [prokaryotic (chloroplast, left panel) and eukaryotic (ER, right panel)] are not independent. Lipids are continuously shuttled between the ER and chloroplast at ER-plastid membrane contact sites. In “C16:3 plants” like Arabidopsis and *Marchantia*, a significant portion of ER-assembled diacylglycerol is returned to the plastid to be incorporated into thylakoid galactolipids, creating a cycle of inter-organelle exchange. Saturated fatty acids (16:0 and 18:0) synthesized de novo in the chloroplast (prokaryotic pathway) are sequentially desaturated by plastid-localized enzymes to yield HTA (16:3n3) and ALA (18:3n3). FAD5 introduces the first double bond into 16:0 to produce 16:1n9, while FAB2 desaturates stearic acid (18:0) to oleic acid (18:1n9). FAD6 subsequently converts both 16:1n9 and 18:1n9 to their Δ^12^ di-unsaturated forms HDA (16:2n6) and LA (18:2n6), and FAD7/8 (*Marchantia* has a single n3-FAD ortholog in the chloroplast) add a third double bond to generate HTA (16:3n3) and ALA (18:3n3). These plastidial n3-PUFAs are channeled through canonical jasmonate biosynthesis to yield OPDA and dn-*cis*-OPDA (common to both species), that are ultimately transformed into JA and JA-Ile (in vascular plants) or dn-*iso*-OPDA (bryophyte-specific). In the ER, FAD2 desaturates 18:1n9 to LA (18:2n6), which is the entry point for the *Marchantia*-specific PUFA elongation cascade. DES6 (Δ^6^-desaturase), Δ^6^-ELO (Δ^6^-elongase), and DES5 (Δ^5^-desaturase) sequentially convert 18:2n6 to ARA (20:4n6). The ER-localized n3-FAD3 then converts ARA to EPA (20:5n3), which feeds the *Marchantia*-specific jasmonate biosynthesis branch to produce C_20_-OPDA and Δ^4^-dn-*iso*-OPDA. Lipids containing EPA may be transported to the chloroplast for further processing. Enzymes conserved between Arabidopsis and *Marchantia* are shown in red; enzymes specific to the *Marchantia* ER pathway are shown in blue. Jasmonate end-products highlighted in yellow are detected only in trace amounts in *Marchantia* or fully absent like JA-Ile; compounds in green dn-*iso*-OPDA, C_20_-OPDA and Δ^4^-dn-*iso*-OPDA are *Marchantia*-specific jasmonates. An asterisk (*) denotes enzymes that have not been fully characterized in *Marchantia*; the curved orange arrow indicates a proposed lipid transport between organelles. DES, Desaturase Enzyme; FAB2, (Stearoyl-ACP Desaturase); FAD, Fatty Acid Desaturase; START2, Steroidogenic Acute Regulatory Protein-Related Lipid Transfer Domain Protein 2. Enzymatic reactions occur on the acyl-esterified forms of fatty acids, primarily esterified to MGDG (chloroplast pathway) or PC (ER pathway).

In angiosperms, the immediate fatty acid substrates for jasmonate biosynthesis are ALA (18:3n3) and, in some species like Arabidopsis and spinach (C_16_ plants), hexadecatrienoic acid (HTA, 16:3n3), produced within the chloroplast or the ER by organelle-specific FADs (e.g., FAD7/FAD8 in Arabidopsis) that act on C_18_ and C_16_ fatty acids esterified to galactolipids in the plastids. Upon diverse stimuli including herbivory and mechanical damage, lipases release these n3-PUFAs (some species produce jasmonates esterified to glycerolipids) to initiate the jasmonate biosynthesis and signaling cascade [8]. In bryophytes, the equivalent C_16_/C_18_ n3-PUFAs are similarly produced, but the enzymology differs in key respects (Figure 2).

The liverwort *M. polymorpha* encodes two n3-FADs, MpFAD3, a microsomal (ER-localized) enzyme equivalent to Arabidopsis AtFAD3, and MpFAD7, a chloroplast-localized enzyme equivalent to AtFAD7/FAD8 [14,27]. Note that MpFAD7 and MpFAD5 (see Section 2.1) are both plastidial enzymes but act at distinct positions on the desaturation cascade; MpFAD5 is a Δ^7^-desaturase introducing the first double bond into 16:0, while MpFAD7 is the terminal n3-FAD converting HDA (16:2n6) and LA (18:2n6) to HTA and ALA, respectively. Cold treatment induces a 3- to 6-fold increase in MpFAD3 transcript levels [27]. Since MpFAD3 catalyzes the conversion of ARA (20:4n6) to EPA (20:5n3) in the ER, this transcriptional upregulation translates into a corresponding increase in EPA content under cold conditions [27]. The cold inducibility of MpFAD3 parallels the cold-induced transcriptional regulation of AtFAD3 in Arabidopsis; however, this regulatory mechanism contributes to EPA (20:5n3) production, a source of *Marchantia*-specific jasmonates (see Section 2.3 below). By contrast, MpFAD7 is only mildly cold-inducible and its disruption has a dramatic effect on canonical (C_16_/C_18_) jasmonate production [14,27].

Recent CRISPR-Cas9-based genetic dissection in *Marchantia* confirmed that MpFAD7 is a plastidial n3-FAD controlling HTA (16:3n3) and ALA (18:3n3) pools that feed the hexadecanoid and octadecanoid jasmonate biosynthetic pathways, respectively. Mutants lacking MpFAD7 are severely depleted in HTA and ALA, and correspondingly impaired in canonical dn-OPDAs and OPDA jasmonate production upon wounding. The ER-localized MpFAD3, in contrast, contributes to the n3-long chain PUFA (n3-LCPUFA) pool specifically by converting arachidonic acid (ARA, 20:4n6) to EPA (20:5n3), and its disruption significantly reduces C_20_-OPDA (a C_20_ OPDA analog derived from EPA) and Δ^4^-dn-*iso*-OPDA (a dn-OPDA analog derived from C_20_-OPDA) production without affecting canonical C_16_/C_18_-derived jasmonates [14]. The Mp*fad3fad7* double mutant hyperaccumulates omega-6 (n6) PUFAs and is a fully jasmonate-impaired line, a crucial tool for understanding jasmonate signaling in an evolutionary context [14].

### 2.3. The C_20_-LCPUFA Pathway: Eicosanoids in Bryophytes

A defining feature that sets bryophyte fatty acid metabolism apart from that of angiosperms is the capacity to synthesize LCPUFAs with chain lengths of C_20_ and beyond [28]. In *Marchantia*, significant quantities of ARA (20:4n6) and EPA (20:5n3) are present, reaching up to 2.2 and 2.6 mg/g dry weight, respectively, in some accessions [28,29]. These LCPUFAs are absent from most angiosperm tissues (apart from very low levels in specialized cells and singular species), making the liverwort lipid composition resemble that of marine algae and lower eukaryotes [13,25,28].

The biosynthetic route from C_18_-PUFAs to LCPUFAs in *Marchantia* was delineated by Kajikawa et al. through heterologous expression and gain-of-function transgenic studies. Linoleic acid (LA, 18:2n6) and ALA (18:3n3) are first desaturated by MpDES6 (Δ^6^-desaturase) to γ-linolenic acid (GLA, 18:3n6) and stearidonic acid (SDA, 18:4n3), respectively. These are then elongated by MpELO1 (Δ^6^-elongase) to dihomo-γ-linolenic acid (DGLA, 20:3n6) and eicosatetraenoic acid (ETA, 20:4n3). Final Δ^5^-desaturation by MpDES5 converts DGLA to AA and ETA to EPA [30]. Thus, EPA biosynthesis in *Marchantia* proceeds through the “Δ^6^-pathway”, a metabolic route shared with many lower eukaryotes, algae, and fungi but distinct from the de novo LCPUFA biosynthesis in marine organisms [31]. This pathway requires all three enzymatic activities (MpDES6, MpELO1, MpDES5) to function coordinately [11]. Angiosperms lack functional Δ^6^-desaturase/elongase and Δ^5^-desaturase activities, which explains why they cannot proceed beyond C_18_-PUFAs under normal conditions [30,31,32]. This metabolic incapacity in flowering plants is consistent with their ancient evolutionary divergence from nonvascular plants in LCPUFA biosynthesis, though whether it represents a primary evolutionary absence rather than a secondary loss has not been definitively resolved by genomic analyses alone [30,31,32]. The expression of bryophyte LCPUFA genes in transgenic *Arabidopsis* was demonstrated to be sufficient for de novo ARA and EPA production *in planta*, confirming the ancestral nature of this pathway [30]. The downstream consequence that EPA serves as an additional substrate for jasmonate biosynthesis in bryophytes was recognized only recently [11,12]. The specific lipase and/or transporter enzyme mobilizing this EPA pool from ER-localized glycerolipids remain uncharacterized (see Section 2.4 and Section 7).

### 2.4. Lipase-Mediated Release of PUFA Substrates from Membrane Glycerolipids

The cleavage of polyunsaturated fatty acids from the *sn-1* and *sn-2* positions of plastidic glycerolipids constitutes an obligatory enzymatic step in jasmonate biosynthesis, providing ALA (18:3n3), HTA (16:3n3), and—uniquely in bryophytes—EPA (20:5n-3) as substrates for lipoxygenase-mediated oxygenation [33,34,35,36,37]. In angiosperms, this lipolytic step is executed redundantly by multiple plastid-localized phospholipase A (PLA)-class enzymes [35]. Historically, DAD1 (a.k.a. PLA-Iβ1) was the first identified plastid-localized acyl hydrolase required for jasmonate-dependent anther dehiscence in Arabidopsis, while a related paralog, DonGLe (PLA-Iα1), was proposed as the primary wound-inducible lipase in leaves [38]. However, independent mutant analyses demonstrated that neither enzyme is individually essential for wound- or pathogen-induced jasmonate accumulation, revealing an unexpectedly broad redundancy within the PLA-I family [35]. A subsequent screen identified PLA-Iγ1 as the single largest individual contributor to wound-induced OPDA, dn-*cis*-OPDA, and JA, though even its disruption only halves wild-type oxylipin levels [35]. More recent work further identified the plastid-localized Plastid Lipase 2 (PLIP2) and Plastid Lipase 3 (PLIP3), both PLA-type enzymes, whose overexpression strongly elevates bioactive jasmonates in an ABA-responsive context [39]. Collectively, these findings have consolidated the view that rapid post-wounding JA accumulation is controlled primarily at the level of post-translational lipase activation rather than de novo transcriptional induction, and that no single lipase is individually rate-limiting in angiosperms [34,36,38,39,40].

A second major lipase family relevant to PUFA mobilization is the Patatin-like Phospholipase (pPLA) superfamily, named for the dominant storage protein of potato tubers and structurally homologous to the Ca^2+^-independent Phospholipase A_2_ (iPLA_2_) of animals. Patatin-domain proteins possess a broad acyl hydrolase activity with no strict *sn*-positional specificity, and the patatin fold is deeply conserved across prokaryotes, fungi, and plants [41]. In Arabidopsis, the pPLA superfamily (subfamilies pPLAI, pPLAII, and pPLAIII) participates in diverse processes including auxin responses and abiotic stress tolerance, but individual members have not been genetically assigned to the jasmonate biosynthetic pathway [42]. Importantly, the *Marchantia* genome encodes a patatin-like phospholipase, Mp5g21760 (*MpPATATIN*), whose transcript is strongly and rapidly induced by mechanical wounding in a COI1-dependent manner [11]. This COI1-dependent wound inducibility places *MpPATATIN* within the same positive-feedback transcriptional loop that regulates other jasmonate biosynthetic genes in *Marchantia*, though its induction downstream of COI1 activation also suggests a role in the amplification phase of PUFA mobilization rather than the immediate initiating event [13]. No genetic loss-of-function analysis of *MpPATATIN* has been reported, and whether it releases free n3-PUFAs from the plastidic galactolipid pool to sustain LOX activity during the wound response remains untested.

Beyond pPLAs, the *Marchantia* genome encodes homologs of secreted phospholipase A_2_ (sPLA_2_) and of phospholipase D (PLD), which generates phosphatidic acid (PA) as a secondary messenger that intersects jasmonate signaling cascades in angiosperms [43]. Notably, PLD gene families in mosses (*P. patens*) form a phylogenetically independent clade from angiosperm PLDs, suggesting lineage-specific functional divergence; however, neither sPLA_2_ nor PLD genes have been functionally characterized in the context of jasmonate biosynthesis or PUFA release in any bryophyte [44,45,46].

The lipolytic step in bryophytes presents additional mechanistic complexity relative to angiosperms. The dominant dn-*iso*-OPDA precursor in *Marchantia* is HTA (16:3n3), esterified primarily at the *sn-2* position of plastidic MGDG; efficient release of HTA therefore requires either a lipase with *sn-2* activity or dual *sn-1*/*sn-2* specificity—a biochemical requirement not formally documented for any identified *Marchantia* lipase candidate. Furthermore, the C_20_-LCPUFA pathway, which produces C_20_-OPDA and Δ^4^-dn-*iso*-OPDA from EPA, is likely initiated in the ER or at the ER-chloroplast interface, consistent with the ER localization of MpFAD3 that synthesizes EPA [11,12,14]. This topological argument predicts that the lipase releasing EPA for the C_20_-jasmonate pathway is a distinct enzyme from the plastid-localized lipase(s) acting on MGDG-esterified HTA—although the possibility that n3-LCPUFA-containing lipids from the ER could get into contact with plastid-localized lipases upon tissue disruption, or via lipid transfer proteins as proposed for MpSTART2 [25] and depicted in Figure 1 and Figure 2, cannot be ruled out at present. Whether *Marchantia* thus employs two lipase activities operating in parallel on different membrane compartments and distinct glycerolipid substrates, mirroring the substrate specialization of its PUFA-generating desaturases, remains a central open question for the field. Resolution of this gap is discussed in Section 7.

## 3. An Expanded Lipoxygenase Family in Bryophytes

### 3.1. LOX Diversity and Their Phylogenetic Position

In Arabidopsis, JA biosynthesis is initiated by 13-LOX enzymes that oxygenate ALA (18:3n3) at the C_13_ position generating (13*S*)-hydroperoxy-octadecatrienoic acid (13-HPOT), the substrate for AOS (Figure 1). As described in the context of Figure 1, in C_16_-plants, such as Arabidopsis and *Marchantia*, HTA (16:3n3) is oxygenated at C_11_ and follows a similar path as ALA (18:3n3). The Arabidopsis genome encodes six LOX genes (AtLOX1-6), of which LOX2, LOX3, LOX4, and LOX6 contribute to wound-induced jasmonate biosynthesis, predominantly through 13-lipoxygenation of ALA [47,48].

**Figure 3:**
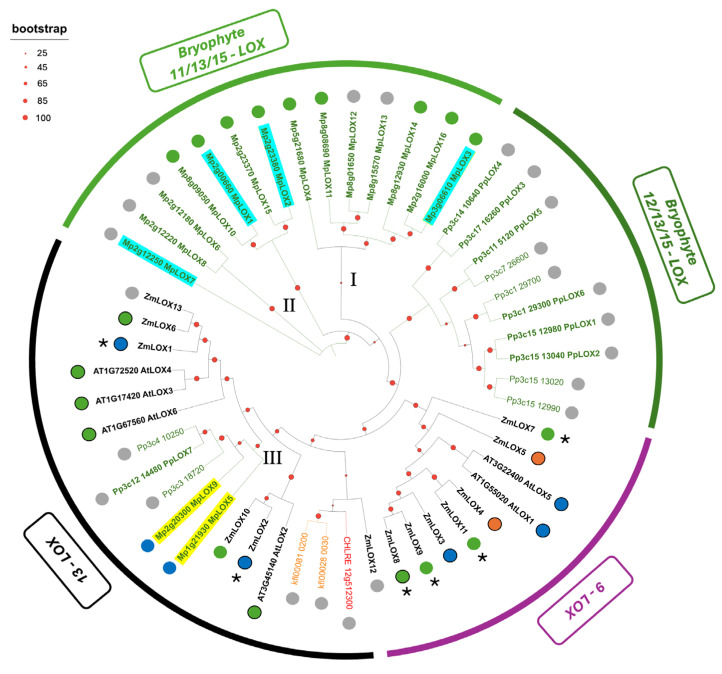
Evolutionary diversification of lipoxygenase (LOX) proteins across Viridiplantae. LOX protein sequences were selected based on a prior orthology analysis in which LOX homologs belonging to the same orthogroup as *M. polymorpha* LOX proteins were identified using OrthoFinder [52]. Protein sequences were aligned using MAFFT [53], and poorly aligned regions were removed with trimAl [54] using the automated trimming option. Maximum-likelihood phylogenetic inference was performed using IQ-TREE3 [55] with automatic model selection using ModelFinder [56], and branch support was assessed using 1000 ultrafast bootstrap replicates. Bootstrap support values are represented by proportional node symbols, with larger circles indicating stronger support. The first letters of protein names designate plant species as follows: At, *Arabidopsis thaliana* (dicots, tracheophytes); CHLRE *Chlamydomonas reinhardtii* (green algae, chlorophytes); kfl, *Klebsormidium nitens* (green algae, charophytes); Mp, *Marchantia polymorpha* (liverworts, bryophytes); Pp, *Physcomitrium patens* (mosses, bryophytes); Zm, *Zea mays* (monocots, tracheophytes). Taxa are color-coded according to major phylogenetic groups: bryophytes (green), spermatophytes (black), charophytes (orange), and chlorophytes (red). The phylogeny highlights lineage-specific diversification of LOX proteins across green plants, with notable expansion and diversification among bryophyte homologs. Roman numbers I, II, and III highlight *Marchantia*’s LOXs clades. Characterized MpLOXs are highlighted in blue. Strong MpLOX candidates for jasmonate biosynthesis are highlighted in yellow. The color of small circles indicates the predicted subcellular location of the LOX protein: green for plastids, blue for cytosolic, orange for tonoplast, and gray for unknown. Circles outlined in black indicate reported experimental evidence. An asterisk (*) next to ZmLOXs indicates the opposite regiospecificity 9-LOX vs. 13-LOX. Regiospecificity and substrate preference inferred from phylogenetic position should always be interpreted with caution.

The lipoxygenase family of enzymes of *M. polymorpha* is both quantitatively larger and qualitatively more diverse (Figure 3) compared to Arabidopsis. The *Marchantia genome* encodes 16 LOX-like sequences, including the four characterized MpLOX1, MpLOX2, MpLOX3 and MpLOX7 proteins [29,49]. Phylogenetic analysis by Kanamoto et al. placed MpLOX1-3 in an independent clade separated from both angiosperm LOX clades (9-LOX and 13-LOX) and from the *P. patens* LOXs [29,49]. This independence reflects the early evolutionary divergence of liverwort LOXs from the ancestor of seed plants and is consistent with the early divergence of the liverwort lineage from other land plants [29,49,50]. A striking biochemical feature of MpLOX1-3 is their strong substrate preference for C_20_-PUFAs. Recombinant enzyme assays demonstrated that EPA and ARA are superior substrates compared with ALA and LA for all three enzymes. Specifically, MpLOX1 exhibits dual 11*S*- and 15*S*-LOX activity against EPA, and 15*S*-LOX activity against ARA, while MpLOX2 and MpLOX3 show predominantly 15*S*-LOX activity against both C_20_-PUFA substrates. Against C_18_ substrates, all three MpLOXs exhibit 13-LOX activity, but this was clearly secondary to the C_20_ preference. The selectivity for 15-PUFA derivatives rather than 13-products has no direct counterpart in angiosperm LOXs and reflects the adaptation of MpLOXs to the liverwort LCPUFA pool [29].

### 3.2. MpLOX7 Accepts C_18_-PUFA Substrates and Contributes to C_6_ Aldehyde Formation

Beyond MpLOX1-3, Tawfik et al. characterized MpLOX7 as a multifunctional enzyme with an unusually broad substrate tolerance, accepting both C_18_ (LA, ALA) and C_20_ (ARA, EPA) PUFAs at comparable efficiency, with ALA showing the highest *k*_cat_ value [49]. This study placed MpLOX7 in a distinct clade of non-seed plant LOXs that encompasses sequences from *Physcomitrium* and *Selaginella* [49]. Its most remarkable property is the capacity to form C_6_ aldehydes (*n*-hexanal from ARA and *Z*-3-hexenal from ALA) as by-products during catalysis, through cleavage of allylic C–C bonds in the hydroperoxide intermediates. This LOX-mediated C_6_ aldehyde formation does not require HPL activity, which *M. polymorpha* lacks entirely at the genomic level [18], and represents an unprecedented enzymatic mechanism for green leaf volatile production in liverworts. This observation could partially explain why *M. polymorpha* can produce C_6_ volatiles like *n*-hexanal and *Z*-3-hexenal despite lacking a canonical HPL gene, offering an alternative evolutionary solution to volatile defense signaling [49]. Whether MpLOX7 is the only *Marchantia* LOX with HPL-like activity and whether it can contribute to jasmonate biosynthesis remain to be elucidated.

### 3.3. Uncharacterized Marchantia LOXs and Jasmonate Biosynthesis

Despite significant recent progress in characterizing MpLOX1–3 and MpLOX7, the specific MpLOX enzyme(s) that catalyze the committed oxygenation step in dn-OPDAs and Δ^4^-dn-*iso*-OPDA biosynthesis *in planta* remain unidentified—one of the most pressing biochemical gap in the entire bryophyte jasmonate pathway. In Figure 3, we present a phylogenetic analysis for LOX enzymes of representative plant lineages and green algae. *Marchantia* LOXs can be divided into three distinct groups (Figure 3, I, II and III). In group I, most MpLOXs possess a chloroplast transit peptide (CTP) and the only enzyme characterized from this clade, MpLOX3, has a predominant 15-LOX activity (see Section 3.1). Group II, including LOX6/7/8, is phylogenetically more distant to seed-plant LOXs than group I and four out of these seven LOXs present a CTP. Three characterized MpLOXs, namely MpLOX1/2/7, fall in group II with MpLOX1/2 and MpLOX7 forming two distinct clades and having diverse substrate preferences (see Section 3.1 and Section 3.2). The third group, group III, is composed of MpLOX5 and MpLOX9, which are clearly placed among seed-plant 13-LOXs, several of which are involved in jasmonate biosynthesis in their respective species. A priori, MpLOX5 and MpLOX9 appear as strong candidates for jasmonate biosynthesis; however, since *Marchantia* produces jasmonates from plastidial C_16_ and C_18_ PUFAs and from ER-produced C_20_ LCPUFAs (requiring 11-, 13- and 15-LOX activity respectively), it is difficult to prioritize LOX candidates based on subcellular localization or substrate preference alone. Nevertheless, cross-stress transcriptomic atlases [51] and positive-feedback wound and jasmonate inducibility data represent important complementary criteria for narrowing the candidate LOX list, alongside CRISPR-Cas9 disruption of prioritized candidates.

## 4. Allene Oxide Synthases in Bryophytes

### 4.1. MpAOS1 and MpAOS2: Two Functionally Diverged CYP74 Enzymes of Marchantia

In jasmonate biosynthesis, the conversion of fatty acid hydroperoxides (the LOX products) into unstable allene oxides is catalyzed by AOS, a member of the cytochrome P450 CYP74 superfamily [57]. In angiosperms, canonical AOS enzymes (classified as CYP74A and CYP74C) show strong preference for 13-hydroperoxides of C_18_ fatty acids, particularly 13-HPOT [58]. Arabidopsis AtAOS (CYP74A) exhibits *K*_m_ values in the low micromolar range for 13-HPOT and is the sole non-redundant AOS required for wound-induced OPDA accumulation [8,57].

*Marchantia* encodes two AOS proteins, MpAOS1 and MpAOS2, which are 59% identical to each other and closely related to *P. patens* PpAOS1 (sharing 49% and 47% identity, respectively) [33,59,60]. MpAOS2 contains a predicted *N*-terminal chloroplast transit peptide of 66 amino acids, suggesting plastid localization and a direct role in dn-OPDA/OPDA biosynthesis in the organelle where the substrate lipids reside. MpAOS1 lacks a predicted organelle-targeting sequence, raising questions about its in vivo localization and function [33,60]. Recombinant enzyme characterization of both MpAOSs revealed a shared preference for 13-HPOT as the best substrate, consistent with other characterized 13-AOSs [60]. However, MpAOS1 displayed unusually broad substrate tolerance, with significant activity not only toward 13-HPOT (100%) but also 15-hydroperoxyeicosapentaenoic acid (15-HPEPE, ~80), 15-hydroperoxyeicosatetraenoic acid (15-HPETE, ~65%), and 9-hydroperoxyoctadecadienoic acid (9-HPOD, ~20%). This acceptance of 15-hydroperoxides of C_20_ PUFAs as substrates is a critical property that might enable MpAOS1 to participate in the C_20_-derived jasmonate pathway [11]. MpAOS2 showed a distinct preference for 13-HPOT and 15-HPEPE over 15-HPETE (ratio of approximately 3:1), indicating a capacity to discriminate between different n3-PUFA-derived hydroperoxides. Neither MpAOS1 nor MpAOS2 showed activity toward 12-HPETE, the proposed precursor of C_8_ volatiles, further supporting that C_8_ volatile formation in *M. polymorpha* requires a separate, as-yet-unidentified enzymatic mechanism [60], likely LOX-mediated. The direct roles of MpAOS1 and MpAOS2 in OPDA biosynthesis were established through CRISPR/Cas9-mediated gene disruption in *Marchantia* [33]. Subcellular localization studies using Citrine fluorescence fusion constructs confirmed that MpAOS1 is cytosol-localized, while MpAOS2 is targeted to the chloroplasts [60]. Notably, single-knockout mutants of either gene alone retained OPDA accumulation; in fact, Mp*aos1* mutants showed elevated OPDA levels relative to wild-type (WT), suggesting that MpAOS2 activity may be compensatorily upregulated when the cytosolic enzyme is absent [33]. Whether this surprising observation implies a coordinated transcriptional regulation or a simple pathway flux redistribution remains to be elucidated. Complete abolishment of OPDA production required disruption of both genes, demonstrating that the cytosolic and chloroplastic pathways contribute redundantly to OPDA accumulation in the thallus. When challenged with the spider mite *Tetranychus urticae*, Mp*aos1aos2* double mutants exhibited significantly higher mite survival rates and increased oviposition compared to WT plants, providing direct evidence of OPDA-mediated defense against mite herbivory [33]. While the study did not quantify dn-OPDAs or Δ^4^-dn-*iso*-OPDA (ligands of MpCOI1 and main jasmonate-dependent mediators of anti-herbivore defense) in either single or double mutants, the pronounced herbivory phenotype strongly suggests that Mp*aos1aos2* plants are expected to be also impaired in the production of these HTA- and EPA-derived jasmonates, respectively. Moreover, the cytosolic localization of MpAOS1 raises the question of how it accesses fatty acid hydroperoxide substrates, which are generated within or in close proximity to the chloroplasts. Two non-mutually exclusive scenarios can be envisioned: (i) hydroperoxide intermediates produced by chloroplast-localized LOX enzymes could be exported to the cytosol (an unexplored but mechanistically plausible lipid trafficking event) and (ii) MpAOS1 may preferentially act on LOX products derived from ER-localized EPA, consistent with its measured activity toward 15-hydroperoxyeicosapentaenoic acid (15-HPEPE, ~80%) and 15-hydroperoxyeicosatetraenoic acid (15-HPETE, ~65%). The demonstrated *in planta* cytosolic localization, combined with the MpAOS1 substrate preference for C_20_-PUFA hydroperoxides, is therefore consistent with a dedicated role in the EPA-derived C_20_-OPDA pathway, whereas MpAOS2 would primarily serve the plastidial HTA/ALA pathway. The compensatory increase in OPDA observed in Mp*aos1* single mutants, where only the chloroplastic enzyme remains active, is consistent with a primary role for MpAOS2 in jasmonate biosynthesis in *Marchantia*, though a direct flux comparison has not been reported [33].

### 4.2. Independent Evolution of Bryophyte CYP74s

One of the most evolutionarily significant findings from the characterization of MpAOSs is the phylogenetic independence of the bryophyte/charophyte CYP74 clade from the canonical angiosperm CYP74 subfamily. Maximum likelihood, neighbor-joining, and Bayesian phylogenetic analyses consistently place MpAOSs, PpAOSs, and the charophyte KfAOS in a single clade that is clearly distinct from the CYP74A (AOS), CYP74B (HPL), and CYP74C (DES) subfamilies found in angiosperms [60]. This evolutionary scenario is consistent with either convergent evolution of AOS activity from distinct ancestral CYP74 proteins, or divergence from a common CYP74 ancestor before the split of bryophyte and angiosperm lineages; distinguishing between these scenarios will require ancestral sequence reconstruction and broader sampling of early-diverging plant genomes [60].

The *P. patens* genome encodes a unique AOS-related HPL (PpHPL) that can catalyze both AOS and HPL reactions on C_18_ and C_20_ substrates, while *Marchantia’s* AOSs lack HPL activity entirely [60]. The charophyte *Klebsormidium flaccidum* contains a single KfAOS with strict preference for 13-HPOs from C_18_-PUFAs, consistent with its lipid composition dominated by LA (18:2n6) over LCPUFAs. Together, these data suggest that the CYP74 gene originally present in the earliest land plants likely had an AOS-like activity and that HPL, DES, and the diversification within the angiosperm CYP74 family occurred later, with HPL and DES activities arising multiple times through convergent evolution from AOS-like ancestors [60].

## 5. Allene Oxide Cyclase (AOC) in Bryophytes

AOC catalyzes the stereospecific ring closure of the unstable allene oxide intermediate generated by AOS, forming the (9*S*,13*S*)-cyclopentenone skeleton that defines the biologically active jasmonates. The enzyme’s eight-stranded antiparallel β-barrel active site enforces exclusive production of the *cis*-(9*S*,13*S*) stereoisomer: a conserved glutamate residue (Glu-23 in AtAOC2; Glu-18 in PpAOC1/2) initiates epoxide ring opening, generating a pentadienyl cation that is stabilized by a catalytic water molecule coordinated by Asn, Ser, and Pro residues, and is then directed to concerted pericyclic ring closure [57,61,62,63]. In the absence of AOC, the same allene oxide intermediate cyclizes spontaneously to a near-equimolar mixture of enantiomers and ketols, underscoring AOC’s role as the indispensable stereochemical gatekeeper committing allene oxide flux to the (9*S*,13*S*)-cyclopentenones required as MpCOI1 ligands in *Marchantia* or as JA-Ile precursors in angiosperms [57,61,62,63].

The genome of Arabidopsis encodes four AOC paralogs (AtAOC1-4) that function as homo- and heterodimers, and show tissue-specific expression patterns [57,61,64]. *M. polymorpha* encodes a single AOC gene, Mp*AOC*, consistent with the low gene redundancy of the jasmonate pathway in this liverwort genome. Recombinant MpAOC produces the naturally occurring (9*S*,13*S*)-OPDA in a coupled in vitro assay with PpAOS1 (used as the AOS reaction partner) and 13-HPOT as substrate, and subcellular localization studies using MpAOC-GFP fusion proteins confirmed chloroplast targeting, mirroring the plastid-localized AOC enzymes of angiosperms [65]. Mp*AOC* expression is strongly induced by wounding and moderately by exogenous OPDA, demonstrating conservation of positive feedback regulation of the jasmonate pathway at the AOC level [65]. Overexpression of MpAOC led to elevated endogenous OPDA levels and growth suppression, directly confirming that OPDA functions as a signaling molecule in the liverwort. Notably, however, the study quantified only OPDA and did not generate Mp*aoc* loss-of-function lines; characterizing the full jasmonate profile, including dn-OPDAs and the Δ^4^-dn-*iso*-OPDA, in both overexpression and knockout lines will be essential to assign MpAOC’s contribution to the complete spectrum of MpCOI1 ligand biosynthesis [65]. The currently untested ability of MpAOC to process C_20_-PUFA-derived allene oxide intermediates has direct implications for the Δ^4^-dn-*iso*-OPDA pathway. If MpAOC cannot cyclize the allene oxide derived from 15-HPEPE (the MpAOS1/2 product from EPA oxygenation), two possibilities arise: (i) an as-yet-undiscovered cyclase operates on C_20_-allene oxides in *Marchantia*, or (ii) the C_20_-OPDA is produced non-enzymatically, a process that would yield a mixture of stereoisomers rather than the pure (9*S*,13*S*) compound. The fact that C_20_-OPDA is detected as a single diastereoisomer *in planta* argues for a stereospecific biosynthesis [11,33].

The genome of *P. patens* encodes two AOC paralogs, PpAOC1 and PpAOC2, both localizing to chloroplasts despite lacking predictable *N*-terminal transit peptides. PpAOC1 shares the substrate specificity of AtAOC2, converting only C_18_-PUFA-derived allene oxides to *cis*-OPDA. PpAOC2, by contrast, accepts both the C_18_-derived allene oxide and the allene oxide from 12-HPETE (a C_20_ ARA-derived substrate), producing the novel cyclopentenone 11-oxo-prostatrienoic acid (11-OPTA) [62]. Crystal structures of PpAOC1 (1.35 Å) and PpAOC2 (1.98 Å) are nearly identical in overall fold to AtAOC2 despite ~450 Myr divergence. However, analysis of the PpAOC2 structure revealed three cooperative features that explain its expanded specificity, (i) a modestly enlarged active site diameter, (ii) an elongated narrow cavity that accommodates the additional carbons of the C_20_ substrate, and (iii) two less bulky residues at the active site entrance that make Arg-22 accessible for electrostatic stabilization of the C_20_ substrate’s carboxylate head group [62]. Site-directed mutagenesis incorporating key PpAOC2 residues into PpAOC1 (F29I/F140V) was insufficient to transfer C_20_-allene oxide activity, indicating that substrate specificity is determined cooperatively by the full active site geometry rather than by any of the tested amino acids [62,66].

Targeted disruption of Pp*AOC1* or Pp*AOC2* in *P. patens* had no effect on vegetative growth under standard conditions but resulted in ~10-fold reduced capsule number, aberrant sporophyte morphology, and arrested sporogenesis at the post-meiotic tetrad stage, suggesting that AOC-dependent cyclopentenone biosynthesis is essential for moss reproduction [66]. Double knockouts were unobtainable in repeated attempts, a result most consistent with, though not proof of, an essential overlapping function between the two paralogs. Exogenous OPDA failed to complement the mutant phenotype, raising the possibility that the biologically active jasmonate in reproductive tissues is not OPDA itself but likely dn-OPDAs, 11-OPTA, or a currently undetected AOC-dependent derivative [66]. These mutant phenotypes, together with the established requirement for JA in angiosperm fertility, suggest that the AOC-dependent oxylipin signaling for sexual reproduction is evolutionarily ancient and predates the full elaboration of the JA-Ile system in vascular plants. The contrast between *P. patens*, where AOC disruption impairs sporophyte development, and *Marchantia*, where jasmonate deficiency does not affect fertility, reflects an important divergence in downstream signaling even within the bryophytes, likely arising from distinct rewiring of the jasmonate co-receptor system across liverwort and moss lineages [62,65,66].

## 6. dn-OPDAs and OPDA Modifications: Isomerization, Conjugation to Amino Acids, Hydroxylation and β-Oxidation on Route to JA

### 6.1. The Cis-to-Iso Isomerization: Paradoxical Fate Divergence

The *cis*-to-*iso* isomerization of dn-OPDA occupies a pivotal position in bryophyte jasmonate biology. While OPDA and dn-*cis*-OPDA are reactive electrophilic species (RES) through their α,β-unsaturated carbonyl group, enabling COI1-independent thermotolerance responses in streptophytes including *M. polymorpha*, *K. nitens*, and Arabidopsis, the *iso* forms lack the RES property due to their more stable tetrasubstituted olefin [67]. Thus, the conversion of dn-*cis*-OPDA to dn-*iso*-OPDA (also valid for the Δ^4^-variants) may stabilize the molecule against non-enzymatic Michael addition by nucleophiles such as glutathione and cysteines in proteins, extending its half-life in the cellular environment and improving its utility as a selective receptor ligand [9,12,67].

In plants, the metabolic fate of dn-*cis*-OPDA diverges. In angiosperms and most ferns, dn-*cis*-OPDA is converted to JA via the β-oxidation pathway (using either OPR3-dependent or OPR3-independent routes), while in bryophytes and lycophytes, it is isomerized to dn-*iso*-OPDA to serve as the primary MpCOI1 ligand [16,17]. Chini et al. observed a striking negative correlation between dn-*iso*-OPDA and JA-Ile levels across representative land plant lineages, with the sole exception of *S. moellendorffii* which synthesizes both at different time scales after wounding [17]. This mutual exclusivity strongly suggests that dn-*cis*-OPDA has two possible metabolic fates, namely isomerization to dn-*iso*-OPDA or β-oxidation toward JA, and that the evolutionary transition between these fates corresponds to the emergence of key enzymes in vascular plant jasmonate signaling.

In pull-down assays, dn-*iso*-OPDA is approximately 100-fold more potent than dn-*cis*-OPDA as a MpCOI1-MpJAZ ligand, indicating that the *iso* isomer is the biologically dominant receptor ligand [9]. The accumulation kinetics after wounding show that dn-*cis*-OPDA appears earlier and then declines as dn-*iso*-OPDA accumulates, consistent with enzymatic conversion from *cis* to *iso*. These trends are also observed when comparing the C_20_-PUFA-derived Δ^4^-dn-*cis*- with Δ^4^-dn-*iso*-OPDAs [9,12]. However, the corresponding plant enzyme responsible for this isomerization is unknown in bryophytes, which may contain high levels of both dn-*iso*- and *iso*-OPDA [11,68]. In *Marchantia*, the isomerization appears to proceed directly from dn-*cis*-OPDA to dn-*iso*-OPDA rather than passing from OPDA through *iso*-OPDA, since *iso*-OPDA is undetectable in the liverwort and Δ^4^-dn-*cis*-OPDA is rapidly converted into Δ^4^-dn-*iso*-OPDA only in the presence of *Marchantia* crude extracts [12,68]. Whether it is a unique liverwort-specific enzyme or a member of an existing protein family recruited to this function are open possibilities that cannot be distinguished without biochemical candidate identification.

Strikingly, *in planta*-produced *cis*-OPDA can be isomerized to *iso*-OPDA in the gut of some phytophagous insects as a detoxification mechanism mediated by glutathione transferases [69,70,71]. This insect-mediated isomerization is presented here not as evidence of a shared enzymatic mechanism, but as a biological precedent demonstrating that *cis*-to-*iso* conversion of OPDA-class molecules is chemically achievable under physiological conditions, a precedent that may inform the search for the corresponding plant isomerase in bryophytes.

### 6.2. Amino Acid Conjugation: A Deactivating Role in Bryophytes

In Arabidopsis, the GH3 family enzyme JAR1 catalyzes the ATP-dependent conjugation of JA to isoleucine to produce the bioactive JA-Ile. This conjugation is the activating step that transforms JA into a potent COI1 ligand [72]. The role of GH3 enzymes in jasmonate metabolism has therefore been considered fundamentally activating in the vascular plant context [8].

In *Marchantia*, the situation is precisely the opposite. Liang et al. identified a family of dn-*iso*-OPDA amino acid (aa) conjugates, including -Glu, -Gln, and -His, that accumulate in response to wounding and herbivory [15]. These conjugates were undetectable in Mp*fad5* mutants that are impaired in dn-*iso*-OPDA biosynthesis, confirming their derivation from the jasmonate pathway. Genetic disruption of the Mp*GH3A* gene via CRISPR-Cas9 abolished dn-OPDA-aa conjugates production and resulted in constitutively elevated levels of the bioactive dn-OPDAs (both dn-*cis*-OPDA and dn-*iso*-OPDA) and enhanced expression of dn-OPDA marker genes even under basal conditions. Correspondingly, Mp*gh3a* mutants showed enhanced sensitivity to exogenous OPDA and significantly elevated resistance to *Spodoptera exigua* larvae compared with wild type [15]. Although biochemical data are absent at present, e.g., in vitro enzyme assays with recombinant MpGH3A, these data strongly suggest that MpGH3A acts as a deactivating enzyme conjugating dn-*iso*-OPDA with amino acids.

The presence of dn-*iso*-OPDA-aa conjugates was demonstrated in other bryophytes (*P. patens*, *Polytrichastrum formosum*) and in the lycophytes *Huperzia selago* and *Selaginella lepidophylla*, but not in the angiosperm Arabidopsis or the charophyte *Klebsormidium nitens*. This phylogenetic distribution mirrors the distribution of dn-*iso*-OPDA itself, suggesting that dn-OPDA conjugate formation is a conserved regulatory mechanism wherever dn-*iso*-OPDA functions as bioactive compound [15]. This metabolic logic is therefore inverted relative to angiosperms. GH3-mediated aa-conjugation of the active jasmonate deactivates the hormone in bryophytes (via MpGH3A acting on dn-*iso*-OPDA), while the equivalent reaction activates the hormone in angiosperms (via AtJAR1/GH3.11 acting on JA to produce JA-Ile). This inversion represents a remarkable example of the same enzymatic reaction class being co-opted for opposite regulatory purposes in different plant lineages and is consistent with the broader theme of radical functional divergence in jasmonate signaling during land plant evolution [15,72,73].

The reversibility of dn-OPDA-aa conjugation remains an open question. If conjugates serve as inactive storage forms that can be hydrolyzed back to dn-*iso*-OPDA by amidohydrolases (analogous to IAA-amino acid hydrolysis by ILR1 in angiosperms), this will constitute a sophisticated buffering mechanism for hormone homeostasis. The *Marchantia* genome encodes a single homolog of angiosperm amidohydrolases, MpILR, that can accept some auxin conjugates as substrates in vitro. Whether MpILR or another hydrolase can act on dn-OPDA-aa conjugates in vivo has not yet been established [74].

### 6.3. Potential Hydroxylation and Other Catabolic Routes

Multiple CYP450 enzymes from the CYP94 family (e.g., CYP94B1, CYP94B3, and CYP94C1) catalyze omega and omega-1 hydroxylation of JA-Ile and JA to produce 12-OH-JA-Ile, 12-OH-JA, 12-COOH-JA-Ile, and related derivatives that modify signaling activity in vascular plants [75,76,77,78,79,80]. These catabolic/regulatory routes are critical for JAZ stability and the duration of jasmonate responses. Notably, 12-OH-JA-Ile itself is a weak COI1 ligand in Arabidopsis, and its accumulation after high JA-Ile conditions may serve as a buffering mechanism [81,82].

Whether equivalent hydroxylation reactions operate on dn-*iso*-OPDA or Δ^4^-dn-*iso*-OPDA in bryophytes is currently unknown. The *M. polymorpha* genome encodes putative CYP94-like sequences, but no functional characterization of these enzymes in the context of dn-OPDA catabolism has been reported. For instance, RNA-seq analysis showed that a gene annotated as MpCYP94L (Mp2g10330) is among the wound-responsive COI1-dependent genes, suggesting it might participate in dn-OPDA metabolism, but its biochemical function remains uncharacterized [11]. Given the growing recognition that dn-OPDA homeostasis involves multiple redundant mechanisms (isomerization, amino acid conjugation, and potentially enzymatic hydroxylation), a thorough characterization of CYP94-like activities in *Marchantia* is warranted.

### 6.4. OPR Enzymes and β-Oxidation in the Bryophyte Jasmonate Context

Bryophytes uniformly lack OPR3. *Marchantia* has only OPR1/2-type (class I) OPRs related to cytosolic OPR1 and OPR2 of Arabidopsis, and the same holds for *P. patens* despite its possession of an OPR3-like sequence (PpOPR3a) that harbors the canonical Phe-His residues needed for cyclopentenone binding but lacks OPR3 enzymatic activity. The molecular basis of PpOPR3a’s catalytic incompetence despite conserved Phe-His residues remains unresolved; possible explanations include differences in the substrate-binding geometry that could prevent cyclopentenone accommodation, or alterations in residues that position NADPH correctly for hydride transfer [83]. The absence of OPR3 activity in all bryophytes tested so far provides strong biochemical evidence that the canonical JA biosynthetic route requiring peroxisomal OPR3 never evolved in these lineages [16].

The fundamental discovery of an OPR3-independent JA biosynthetic pathway in *Arabidopsis* revealed that OPDA can bypass OPR3 by undergoing successive β-oxidation cycles to produce 4,5-didehydro-jasmonic acid (4,5-ddh-JA), which is then reduced to JA by the cytosolic OPR1 and OPR2 [16] (Figure 1). Phylogenetic analysis of OPR genes across the land plant lineage confirmed that OPR1/OPR2 class enzymes are conserved in all streptophytes, including charophytes, whereas OPR3 function appears only in vascular plants [16]. In 2017, Han proposed the evolutionary primacy of the OPR3-independent pathway [84], and the broad oxylipin profiling by Chini et al. confirmed it: 4,5-ddh-JA and JA co-accumulate across virtually all streptophyte lineages, with OPR1/OPR2 orthologs present even in the chlorophyte *Chlamydomonas reinhardtii* [16,17,18].

In the bryophyte context, this means that any residual JA detected in this phylum likely results from dn-*cis*-OPDA/OPDA β-oxidation via the OPR1/2 route, functioning as a degradation pathway rather than a hormonal biosynthetic pathway on route to JA-Ile. The failure of exogenous JA to elicit any detectable growth inhibition in *Marchantia*, *P. patens*, or the hornwort *Anthoceros agrestis* confirms that JA is physiologically inert in these species [17]. The independent emergence of OPR3 in vascular plants correlates with the appearance of a JA-Ile-based signaling system, and the peroxisomal channeling of OPDA and dn-*cis*-OPDA toward JA by OPR3 may have been a prerequisite for (or co-evolved alongside) this metabolic transition during land plant evolution.

## 7. Summary Table, Current Gaps and Open Questions in Bryophytes’ Jasmonate Biosynthesis

Jasmonate biosynthesis in bryophytes represents a fascinating evolutionary snapshot of a receptor-coupled oxylipin signaling system in a state prior to the emergence of the canonical angiosperm JA-Ile pathway. The pathway shares its fundamental enzymatic architecture, i.e., lipolytic n3-PUFA release, LOX-mediated oxygenation, AOS cyclization initiation, AOC stereospecific ring closure, but is distinguished from the angiosperm equivalent in several aspects: (i) additional n3-LCPUFA input from EPA via ER-localized MpFAD3; (ii) inverted relative importance of the hexadecanoid versus octadecanoid pathways, with HTA (16:3n3) providing the dominant dn-*iso*-OPDA precursor; (iii) a phylogenetically independent evolution of CYP74 AOSs with expanded substrate range; (iv) evidence for a predicted double-bond isomerase deactivating the RES capacity of the canonical cyclopentenone dn-*cis*-OPDA; (v) the absence of functional OPR3 and JAR1, defining the metabolic endpoint as dn-*iso*-OPDA rather than JA-Ile; and (vi) the deactivating (rather than activating) role of GH3-mediated amino acid conjugation. Table 1 summarizes all aspects of jasmonate biosynthesis in bryophytes discussed here.

Despite the rapid progress of the last decade, several major gaps in understanding remain around jasmonate biosynthesis in bryophytes. The identity of the lipase(s) releasing HTA, ALA, and EPA from membrane glycerolipids in *Marchantia* remains uncharacterized (see Section 2.4 for full discussion). The patatin-like candidate Mp*PATATIN* (Mp5g21760) is wound-induced and COI1-dependent, and its CRISPR-Cas9 disruption paired with comprehensive oxylipin profiling represents the most tractable near-term approach. Moreover, the MpFAD landscape has not been genetically addressed in full. For instance, the individual contribution of MpFAD6 to HTA (16:3n3) and derived jasmonate biosynthesis in *Marchantia* remains to be elucidated. MpFAD6 is predicted to catalyze the desaturation of HD (16:1n9) to HDA (16:2n6), an obligate step in the MpFAD5-dependent pathway to HTA. Loss-of-function mutants of MpFAD6 would complement the existing MpFAD5 and MpFAD7 mutant toolkit and are a logical next step.

The LOX(s) enzyme that directly contributes to jasmonate production in bryophytes remains elusive. No genetic mutants have been used to demonstrate which LOX(s) contribute to either jasmonates, C_6_ or C_8_ volatiles in vivo [49]. Thus, resolving these questions is foundational to understanding the full breadth of LOX-mediated oxylipin signaling in liverworts.

The in vivo functional contributions of MpAOS1 versus MpAOS2 have not been fully resolved genetically. Their differential substrate specificities (MpAOS2 preferring C_18_ hydroperoxides and MpAOS1 accepting both C_18_ and C_20_ hydroperoxides) hint at specialized roles in canonical versus n3-LCPUFA-derived jasmonate pathways. However, knockout mutants have been only characterized for their OPDA producing capacity while the full spectrum of MpAOS1/2-dependent jasmonate production remains to be elucidated [33,60].

The identity of the enzyme responsible for the *cis*-to-*iso* isomerization of dn-OPDA remains one of the most important unresolved questions in bryophyte jasmonate biology. This enzyme is rate-limiting for production of the most potent MpCOI1 ligand, yet no candidate gene has been identified. Whether the same isomerase operates on both dn-*cis*-OPDA and Δ^4^-*cis*-dn-OPDA is also unknown. Likewise, the potential role of omega/omega-1 hydroxylation by CYP94-like enzymes in dn-OPDA catabolism remains completely uncharacterized in *Marchantia*. Given the importance of these reactions in JA-Ile modulation/inactivation in angiosperms, their characterization in bryophytes could reveal whether CYP94-mediated oxidation is an evolutionarily conserved feature of jasmonate catabolism. Moreover, whether the reversibility of dn-OPDA-aa conjugation constitutes a genuine homeostatic mechanism (in analogy to auxin conjugate hydrolysis) or whether most conjugates are irreversibly inactivated remains to be determined experimentally [15].

Another underappreciated gap, not directly part of the biosynthetic cascade but critical to understanding hormone activity, concerns the subcellular transport of bryophyte jasmonates. In Arabidopsis, JAT1 transports JA-Ile into the nucleus and JAT2 exports JA from the peroxisome to the cytosol (see Figure 1). *Marchantia* encodes three putative JAT1 orthologs, but none has been experimentally validated [18]. Whether dn-*iso*-OPDA and Δ^4^-dn-*iso*-OPDA use JAT-like transporters for nuclear entry, given their distinct carbon-chain lengths compared to JA/JA-Ile, remains completely unexplored.

The LCPUFA jasmonate pathway is specifically important in the context of biotechnology and needs further investigation. The expression of bryophyte desaturase and elongase genes in oilseed crops to produce EPA-enriched seeds might introduce a C_20_-OPDA/Δ^4^-dn-*iso*-OPDA biosynthetic capacity with unknown agronomic and ecological consequences [11,14]. Broader metabolomic and genomic comparisons across the ~20,000 bryophyte species could reveal further diversity in jasmonate pathway configurations [86].

Finally, systematic studies of jasmonate biosynthesis in hornworts (Anthocerotophyta) are almost entirely lacking, despite their phylogenetically pivotal position as potentially the sister group to vascular plants. The one published functional study showed that *A. agrestis* is insensitive to exogenous JA, consistent with a dn-OPDA-based signaling system, but the upstream biosynthetic pathway has not been genetically dissected in any hornwort [17]. Extending the detailed biochemical and genetic analysis of jasmonate biosynthesis to *A. agrestis* and additional liverwort species would establish whether the features described for *Marchantia* are universal across bryophytes or specific to the liverwort lineage.

## 8. Conclusions and Perspectives

Jasmonate biosynthesis in bryophytes represents a fascinating evolutionary snapshot of a receptor-coupled oxylipin. The past decade of research on *Marchantia* has fundamentally reshaped our understanding of how jasmonate signaling evolved in land plants. The emerging picture reveals a pathway that is neither a simplified precursor of the angiosperm system nor a mere evolutionary curiosity, but rather a functionally sophisticated and biochemically distinctive oxylipin signaling network that solved the challenge of stress-responsive hormone regulation through mechanisms substantially different from those operating in flowering plants.

Two features stand out as the most evolutionarily significant contributions of bryophyte jasmonate biology. The first is the *cis*-to-*iso* isomerization of dn-OPDA, which constitutes a bryophyte-specific ligand activation mechanism that is chemically and enzymatically distinct from the JAR1-mediated JA activation via conjugation in vascular plants. The conversion of dn-*cis*-OPDA to dn-*iso*-OPDA generates a more potent MpCOI1 ligand, and eliminates the RES character of the cyclopentenone ring, thereby decoupling COI1-dependent receptor signaling from COI1-independent electrophilic responses, a step achieved by OPR3 in vascular systems. The second defining feature is the inverted role of GH3-mediated amino acid conjugation: whereas JAR1/GH3.11 activates the hormone in angiosperms by producing JA-Ile, MpGH3A deactivates the hormone in *Marchantia* by conjugating dn-*iso*-OPDA to amino acids such as glutamate, glutamine, and histidine. This functional inversion of the same enzyme class for opposite regulatory purposes in different plant lineages is one of the most compelling examples of biochemical co-option in plant hormone evolution; its conservation across bryophytes and early-diverging lycophytes pinpoints it as the ancestral state prior to the emergence of JA-Ile-based signaling.

Taken together with the absence of functional OPR3 and JAR1 orthologs in all bryophytes examined to date, and the phylogenetically independent evolution of the bryophyte CYP74 clade, a coherent picture emerges: the shared scaffold of the jasmonate pathway (lipolytic n3-PUFA release, LOX oxygenation, AOS/AOC processing, and COI1/JAZ/MYC perception) was already established before the divergence of bryophyte and vascular plant lineages, while the specific chemistry of ligand activation and inactivation evolved independently within each lineage. The *Selaginella* intermediate, which accumulates both dn-*iso*-OPDA and JA-Ile, marks the approximate phylogenetic window during which this transition occurred and represents an invaluable system for future comparative studies.

Despite this progress, three experimental gaps are particularly interesting. The identification of the *cis*-to-*iso* isomerase is arguably the most pressing priority: this enzyme is rate-limiting for the production of the dominant MpCOI1 ligand and yet remains entirely unknown at the molecular level. Concrete strategies for its identification should include (i) activity-guided fractionation of *Marchantia* protein extracts using dn-*cis*-OPDA as substrates; (ii) comparative transcriptomics to identify wound/COI1-induced candidates with unknown function; and (iii) heterologous expression and in vitro biochemical validation. Second, the identity of the LOX enzyme(s) that catalyze the committed oxygenation step in dn-OPDA biosynthesis *in planta* remains unknown despite the biochemical characterization of four MpLOX proteins. Systematic CRISPR-Cas9 disruption of prioritized candidates represents the most tractable path forward. Third, the functional dissection of lipase-mediated PUFA release in *Marchantia* remains incomplete. The patatin-like phospholipase MpPATATIN is the leading genetic candidate for the amplification phase of PUFA mobilization during wounding, and CRISPR-Cas9 loss-of-function analysis paired with comprehensive oxylipin profiling is long overdue. Whether a separate, ER-associated lipase acts in parallel to release EPA for the C_20_-jasmonate branch remains an open question that connects directly to the mechanism of ER-to-chloroplast lipid trafficking, likely via MpSTART2.

Beyond *Marchantia*, extending the genetic and metabolic dissection of jasmonate biosynthesis to hornworts will be essential for determining which features of the bryophyte pathway are universal across non-vascular land plants and which are liverwort-specific innovations. Similarly, a complete genetic toolkit for *P. patens* jasmonate biosynthesis, including the unresolved roles of PpOPR3a and PpAOS/HPL bifunctional enzyme, would strengthen comparative conclusions currently drawn predominantly from a single model system. Resolving these gaps has significance beyond bryophyte biology. The bryophyte jasmonate biosynthetic pathway provides an evolutionary window into the ancestral oxylipin signaling system from which the angiosperm JA-Ile pathway ultimately derived. Understanding the molecular logic in *Marchantia* will clarify which enzymatic innovations were necessary for the transition to jasmonate signaling in vascular plants—information directly relevant to the rational engineering of stress tolerance and defense responses in crops. Furthermore, the bryophyte-specific capacity to channel C_20_-LCPUFAs into jasmonate biosynthesis introduces a metabolic dimension entirely absent from flowering plant models. Its biotechnological implications, particularly in the context of engineering EPA-accumulating oilseed crops, warrant careful evaluation of the signaling consequences that may accompany altered PUFA profiles. In this sense, *Marchantia* is not only a window into the evolutionary past of jasmonate signaling, but also a forward-looking model for understanding full biosynthetic and regulatory networks in land plants.

## Figures and Tables

**Table 1:** Summary of characterized and uncharacterized enzymes potentially involved in jasmonate biosynthesis in *Marchantia polymorpha*.

Enzyme ^#^	Gene ID	Arabidopsis Ortholog	Subcellular Localization	Reaction Catalyzed	Key Evidence	Mutant *
MpFAD5	Mp3g10660	FAD5	Chloroplast (predicted)	Δ^7^-desaturation of 16:0 → 16:1n9	Genetic: Mp*fad5* nearly abolishes dn-OPDA after wounding [13].	Yes (CC) ^a^
MpFAD6 (FAD10)	Mp4g24020	FAD6	Chloroplast (predicted)	Δ^12^-desaturation of 16:1(n9) → 16:2(n6) (HDA); obligate step toward HTA	Inferred by pathway logic; no functional study reported.	No
MpFAD7 (FAD11)	Mp5g20700	FAD7/FAD8	Chloroplast (confirmed)	n3-desaturation of HDA (16:2n6) → HTA (16:3n3) and LA (18:2n6) → ALA (18:3n3)	Genetic: Mp*fad7* depleted in HTA/ALA and canonical jasmonates [14].	Yes (CC) ^b^
MpFAD3 (FAD8)	Mp3g17690	FAD3	ER (confirmed)	n3-desaturation of ARA (20:4n6) → EPA (20:5n3)	Genetic: Mp*fad3* depleted in EPA and abolishes C_20_-OPDA and Δ^4^-dn-*iso*-OPDA [14].	Yes (CC) ^b^
MpDES6	Mp6g17330	None (bryophyte-specific)	ER (predicted)	Δ^6^-desaturation: LA (18:2n6) → GLA (18:3n6); ALA → SDA (18:4n3); entry into LCPUFA elongation	Biochemical: heterologous expression in yeast/plants [85,30]. Genetic: Mp*des6* depleted in EPA and abolishes C_20_-OPDA and Δ^4^-dn-*iso*-OPDA [11].	Yes (CC) ^a^
MpELO1	Mp6g02350	None (bryophyte-specific)	ER (predicted)	Δ^6^-elongation: GLA (18:3n6) → DGLA (20:3n6); SDA (18:4n3) → ETA (20:4n3)	Biochemical: heterologous expression in yeast/plants [85,30].	No
MpDES5	Mp3g13250	None (bryophyte-specific)	ER (predicted)	Δ^5^-desaturation: DGLA (20:3n6) → ARA (20:4n6); ETA (20:4n3) → EPA (20:5n3)	Biochemical: heterologous expression in yeast/plants [85,30]. Genetic: Mp*des5* depleted in EPA. Abolishes C_20_-OPDA but not Δ^4^-dn-*iso*-OPDA [11].	Yes (CC)
MpPATATIN	Mp5g21760	PLA1 (DAD1)	Unknown	Putative lipase releasing n3-PUFAs from membrane glycerolipids	Transcriptomic: wound-induced, COI1-dependent [11,13]	No
MpLOX1	Mp2g00660	None (liverwort-specific clade)	Unknown	11*S*/15*S*-LOX activity on EPA/ARA; secondary 13-LOX on C_18_-PUFAs	Biochemical: recombinant enzyme assays [29].	No
MpLOX2	Mp2g23380	None (liverwort-specific clade)	Unknown	15*S*-LOX activity on EPA/ARA; secondary 13-LOX on C_18_-PUFAs	Biochemical: recombinant enzyme assays [29].	No
MpLOX3	Mp3g06610	None (liverwort-specific clade)	Unknown	15*S*-LOX activity on EPA/ARA; secondary 13-LOX on C_18_-PUFAs	Biochemical: recombinant enzyme assays [29].	No
MpLOX7	Mp2g12250	Non-seed-plant LOX clade	Unknown	C_6_ aldehyde (*n*-hexanal, *Z*-3-hexenal) formation via HPL-independent mechanism	Biochemical: recombinant enzyme assays [49].	No
MpAOS1	Mp3g21350	CYP74 (independent clade)	Cytosol (confirmed)	AOS activity on 13-HPOT, 15-HPEPE, and 15-HPETE; likely participates in C_20_-jasmonate pathway	Genetic: Mp*aos1 aos2* double mutant abolishes OPDA; single *Mpaos1* shows elevated OPDA [33,60].	Yes (CC) ^c^
MpAOS2	Mp5g16260	CYP74 (independent clade)	Chloroplast (confirmed)	AOS activity; primary preference for 13-HPOT and 15-HPEPE; likely main contributor to canonical dn-OPDA	Genetic: see MpAOS1; Mp*aos2* single mutant retains OPDA [33,60].	Yes (CC) ^c^
MpAOC (DIR17)	Mp7g06220	AOC1-4	Chloroplast (confirmed)	Stereospecific ring closure of allene oxide → (9*S*,13*S*)-dn-*cis*-OPDA or OPDA.	Biochemical + overexpression: elevated OPDA, growth suppression; C_20_-allene oxide activity untested [65].	No
*cis*-to-*iso* isomerase	Unknown	None identified	Unknown	Conversion of dn-*cis*-OPDA → dn-*iso*-OPDA (and Δ^4^-dn-*cis*- → Δ^4^-dn-*iso*-OPDA)	Enzymatic activity in crude extracts [12]; no candidate gene identified	No
MpGH3A	Mp6g07600	JAR1/GH3.11	Unknown	Amino acid conjugation of dn-*iso*-OPDA → dn-OPDA-Glu/-Gln/-His (deactivating step)	Genetic: Mp*gh3a* abolishes conjugates, elevates dn-OPDAs, enhances defense [15].	Yes (CC)
MpILR1	Mp1g20090	IAR3/ILL6 (auxin hydrolase)	Unknown	Amidohydrolase; possible regeneration of free dn-*iso*-OPDA from aa-conjugates	In vitro activity on some auxin-aa conjugates; dn-OPDA conjugate hydrolysis untested [74].	No
MpCYP94s	Mp2g10330	CYP94B/C (hydroxylases)	Unknown	Putative ω-/ω-1 hydroxylation of dn-*iso*-OPDA and Δ^4^-dn-*iso*-OPDA	Transcriptomic: wound-induced, COI1-dependent [11]; no biochemical data	No
MpOPR1/MpOPR2	Mp4g01180/Mp6g05600	OPR1/OPR2	Unknown	Reduction of 4,5-ddh-JA; residual JA biosynthesis	No genetic or biochemical characterization; OPR3 activity absent [16,17].	No

^#^ names in brackets indicate the actual name in https://marchantia.info/. * CC, CRISPR-Cas9-generated mutant. A superscript letter indicates availability of a double mutant of this enzyme and another enzyme sharing the same superscript letter.

## Data Availability

All data and conclusions are derived directly from the primary sources cited herein.
